# “Catheter replacement in catheter-associated urinary tract infection: current state of evidence “

**DOI:** 10.1007/s10096-024-04878-9

**Published:** 2024-06-25

**Authors:** Annette C. Westgeest, Janneke I.M. van Uhm, Laura Pattacini, Wouter Rozemeijer, Barbara M.A. Schout, Rolf H.H. Groenwold, Suzanne E. Geerlings, Merel M.C. Lambregts

**Affiliations:** 1 Department of Infectious Diseases, Leiden University Medical Center, Albinusdreef 2, Leiden, 2333ZA Netherlands; 2 Department of Urology, Leiden University Medical Center, Leiden, Netherlands; 3 Department of Medical Microbiology, Northwest Clinics, Alkmaar, Netherlands; 4 Department of Urology, Alrijne Ziekenhuis, Leiderdorp, Netherlands; 5 Department of Clinical Epidemiology, Leiden University Medical Center, Leiden, Netherlands; 6 Department of Internal Medicine, Division Infectious Diseases, Amsterdam Institute Infection and Immunity, Amsterdam, Netherlands

**Keywords:** Urinary tract infection, Catheter related infection

## Abstract

**Purpose:**

Catheter associated urinary tract infection (CAUTI) is the most common healthcare associated infection. A significant knowledge gap exists regarding the necessity of catheter replacement as part of CAUTI treatment. Current guidelines recommend replacement for faster recovery and to prevent recurrences, but adherence is low. In this systematic review, we aimed to assess the available evidence regarding catheter replacement for CAUTI.

**Materials and methods:**

Eligible studies investigated the effect of catheter replacement in CAUTI on clinical outcomes and/or recurrence rates, irrespective of catheter type or setting. We searched electronic literature databases from inception to October 15th, 2023. Information was extracted regarding setting, eligibility criteria, definition of CAUTI, timing of replacement, and outcomes.

**Results:**

Of the 257 identified studies, four were considered relevant and included. Two were randomized controlled trials (RCT) and two were observational studies. One RCT showed higher rates of clinical recovery and lower recurrence rates in the replacement group, while results of the other RCT favoured retainment, with a lower recurrence rate in the retainment group, although longer antimicrobial treatment in this group. Two observational studies were inconclusive.

**Conclusions:**

Current guidelines rely heavily on recommendations from a single study, emphasizing the need for further research. The burden of catheter replacement, including patient discomfort and resource impact, warrants careful consideration. A randomized trial is essential to provide more evidence on the effect of catheter replacement on clinical outcomes including CAUTI recurrence.

**Supplementary Information:**

The online version contains supplementary material available at 10.1007/s10096-024-04878-9.

## Introduction

The cornerstone of catheter associated urinary tract infection (CAUTI) treatment is antibiotic therapy. One significant knowledge gap pertains to whether it is necessary to replace the catheter as part of the treatment for CAUTI. In case of obstruction or malposition of the catheter, there is consensus — the catheter needs to be replaced. However, for a well-functioning catheter, the debate is more contentious. Current guidelines recommend to change the catheter if it has been in place for longer than two weeks [1, 2]. This is believed to result in faster recovery, a shorter duration of symptoms, and a lower risk of recurrent CAUTI. However, this recommendation is based on limited evidence.

The effectiveness of catheter replacement in mitigating symptoms or preventing recurrent CAUTIs is a topic of ongoing debate. While guidelines suggest that routine catheter replacement may reduce the risk of re-infection, others argue that this practice may be unnecessary, costly, and potentially harmful to patients due to the risks associated with catheter removal and insertion.

The aim of this systematic review was to assess the available evidence regarding catheter replacement for CAUTI on recurrence of CAUTI and clinical outcome.

## Methods

This systematic review was reported in accordance with the *Preferred Reporting Items for Systematic reviews and Meta-analyses* (PRISMA) 2020 guidelines [3]. Details of the protocol for this systematic review were registered at PROSPERO (CRD42023467230).

We included studies investigating the effect of catheter replacement in patients with CAUTI on duration of symptoms and/or recurrence of urinary tract infection (UTI). Eligible studies encompassed various catheter types, including transurethral, suprapubic, nephrostomy, and JJ-catheters. Inclusion criteria extended to all adult patients, including those who were immunocompromised. Additionally, all time points for catheter replacement—whether before the initiation of antibiotic therapy or during antibiotic therapy—were considered for inclusion.

To be included, a study had to report on either recurrence of UTI, duration of symptoms, clinical cure rate, length of hospital stay and/or mortality. Studies that were limited to asymptomatic bacteriuria were excluded. To avoid language bias, studies published in non-English language journals were eligible for inclusion if one of the investigators could read the foreign language (French, Italian, Spanish, German and Dutch). All study settings (community, outpatient and inpatient) were allowed.

We searched multiple electronic databases: PubMed, Embase, Web of Science, the Cochrane Library, and Academic Search Premier, clinicaltrials.gov from inception to October 15th 2023. Our search strategy, constructed by an experienced librarian and based on a PICO-style approach, is provided in Supplement 1. Next, we carried out a ‘snowball’ search to identify additional studies by searching reference lists of study reports included in this systematic review. We did not apply any filters regarding date of publication. Before submission of this review, a search update was conducted to identify recently published studies.

References were imported into Covidence software [4]. Title and abstract screening, as well as full-text screening, was performed independently by two reviewers (ML, AW). In case of disagreement, consensus was reached by discussion between the two researchers. In case of persisting disagreement, a third researcher was consulted (JvU). For each study that was selected for data extraction, the following information was collected: study design, eligibility criteria, population characteristics, number of participants, type of catheter, definition of CAUTI, recurrence of UTI (and definition), duration of symptoms (and definition), clinical cure rate (and definition), mortality rates, length of hospital stay, ICU-admittance, complications of catheter replacement, and duration of follow-up. For each paper, data extraction was performed independently by two reviewers (LP and ML). In case of disagreement a third reviewer was consulted (AW).

The Newcastle Ottawa Scale was used for assessing risk of bias in cohort studies and the RoB tool for assessing bias in randomized trials [5, 6]. 

## Results

We identified 257 potentially eligible studies. After removal of duplicates, we screened 253 titles and abstracts. Most studies were excluded because they applied catheter replacement to prevent CAUTI, e.g., after surgery. We then reviewed 12 full text articles. At this stage, 8 reports were excluded that either did not include our target population, e.g., patients without CAUTI, or applied catheter replacement for other indications. Finally, a total of four studies were included [7–10]. The study selection process is summarised in a PRISMA flowchart (Fig. S2). We identified no protocols of planned or currently ongoing trials.

### Study characteristics, settings and results

The selected papers describe the results of two randomized controlled clinical trials (RCT) [7, 8], of which one has a non-inferiority design, [8] and two observational studies [9, 10]. The settings include medical centers, geriatric centers, and a university medical center. The protocols of the RCTs were not published prior to initiation of the studies. Patients included in the studies had a long-term catheter and were limited to suprapubic and transurethral catheters. We identified no studies in patients with percutaneous nephrostomy catheters or JJ-catheters. There was heterogeneity in the definition of CAUTI (Table 1), but in three out of four studies the definition included clinical symptoms. Studies also differed with regard to disease severity, in two studies signs of systemic inflammation were mandatory for inclusion. [7, 10]. The studies are summarized in Table 2.

**Table 1:** Definition of catheter associated urinary tract infection in the 4 included studies

	Clinical	Microbiological	Pyuria
Kumazawa	Catheter for ≥ 21 days	> 10^4^ CFU/ml	> 5 WBC/HPF
Raz	Indwelling catheter Fever or hypothermia, other signs of infection (leukocytosis or leukopenia)	> 10^3^ (one organism) or 10^4^ (two organisms) CFU/ml	
Darouiche	Indwelling catheter ≥ 1 symptom: fever, suprapubic or flank discomfort, bladder spasm, increased spasticity, worsening dysreflexia and cloudy urine	> 10^5^ CFU/ml	> 10 WBC/HPF
Babich	Catheter for ≥ 7 days Systemic inflammatory response syndrome (SIRS) Clinical exam and chest imaging to exclude other causes of infection	> 10^3^ (one organism) or 10^4^ (two organisms) CFU/ml or bacteremia caused by an uropathogen Bacteremia caused by an uropathogen	> 10 WBC/ml

*Legend* WBC/HPF: white blood cells/high-power field. CFU: colony forming units

**Table 2:** Summary of included studies

Author	Design	Setting and study population	Number of participants	Intervention	Resolution of symptoms/ clinical efficacy	30-day mortality	Recurrence rate
Kumazawa 1992	Observational cohort study	Transurethral catheters > 21 days	Total 56 29 replacement 27 retainment	Catheter replacement before start of antimicrobial therapy (intervention group) Retainment of the catheter (control group) Antimicrobial therapy 300 mg or 600 mg levofloxacin.	Clinical efficacy (not defined) 14/29 with catheter replacement (48%) 14/27 without replacement (52%)	Not reported	Not reported
Raz 2000	Randomized, controlled trial	Long-term care facilities, chronic indwelling urethral catheter	Total 54 27 replacement 27 retainment	Catheter replacement before start of antimicrobial therapy (intervention group) Catheter retainment (control group) In both groups empirical antibiotic treatment with a quinolone and total treatment duration 14 days.	In the replacement group time to resolution of fever was shorter (log rank test t 5 3.247, 0.0.10 p 0.0.05) Cure/improvement at 72 h 25/27 (93%) in the replacement group versus 11/27 (41%) in the retainment group Clinical outcome one week after antibiotic treatment similar in both groups At day 28, 24 participants (89%) randomized to catheter replacement remained cured/improved compared to 16 (54%) without replacement (*p* < 0.001).	0/27 in the replacement group versus 2/27 in the retainment group. Cause of death: urosepsis	28-day recurrence rate was 3/27 in the replacement group and 7/27 in the retainment group.
Darouiche 2014	Randomized, controlled, non-inferiority trial	Hospitalized patients with spinal cord injury 2007–2011. Suprapubic or transurethral catheters. Patients with sepsis were excluded	Total 55 28 replacement 27 retainment	5-day regimen of antibiotics and catheter exchange before start of antibiotic therapy (experimental group) 10-day regimen of antibiotics with catheter retention (control group)	Clinical cure at end of therapy 100% in both groups	2/28 in the replacement group versus 1/27 in the retainment group	9/28 in the replacement group and 3/27 in the retainment group
Babich 2018	Observational prospective cohort study Propensity score matching	Hospital setting, tertiary care hospital, adults with ˃7 days indwelling transurethral catheter 2010–2015, Patients with systemic inflammatory response.	Total 245 89 replacement 156 retainment	Catheter replacement within 6 h after admission or blood withdrawal for sepsis/suspected UTI (intervention group) No catheter replacement (control group)	Clinical failure (death or sepsis at day 7) OR 0.90 (95% CI 0.50–1.63) Resolution of fever at day 7 58/89 (65%) in the replacement group versus 121/154 in the retainment group. (79%)	OR 0.76, 95% CI 0.40–1.44	Not reported

Kumazawa and Matsumoto describe the results of a prospective observational cohort study designed to test the efficacy of two different antibiotic regimens (levofloxacin 300 mg versus levofloxacin 600 mg) with or without replacement of the urethral catheter [9]. Catheter replacement was timed before start of antimicrobial treatment, and treatment efficacy was defined as resolution of bacteriuria and pyuria. In this study, published in 1992, 56 patients were categorized in four groups. Patients who received catheter replacement were more often treated with high dose antimicrobial therapy (17/29) compared to patients without catheter replacement (10/27). The study failed to show any difference in resolution of bacteriuria and pyuria. Follow-up time was not described and the recurrence rate of CAUTI was not reported.

In 2000, Raz and coworkers published the results of an RCT that enrolled 54 long-term residents of two nursing homes, who had transurethral catheters and presented with acute CAUTI with systemic symptoms [11]. Patients with catheter obstruction or gross hematuria were excluded. Patients were randomized into catheter replacement before initiation of antimicrobial therapy or retainment of the catheter, and all patients were empirically treated with a quinolone. The results of this study showed a higher rate of microbiological resolution (negative culture) in the replacement group at 72 h after start of therapy, as well as at 7 and 28 days after therapy discontinuation. Clinical cure was similar between the groups at 7 days after therapy (25/27 [93%] in the replacement group and 21/27 [78%] in the retainment group). However, after 28 days, the rate of clinical cure was higher in the replacement group with 24/27 (89%) remaining cured versus 15/27 (56%) in the retainment group. The replacement group had a lower recurrence rate (3/27 [11%] versus 7/27 [26%]) as measured 28 days after therapy termination. Two patients in the no-replacement group died of urosepsis within three days after start of therapy.

Darouiche and coworkers aimed at proving the non-inferiority of catheter replacement and 5 days of antibiotic treatment as compared to 10 days of antibiotic treatment with retainment of the catheter [8]. The study was conducted at a veterans affairs medical center and enrolled patients with spinal cord injury from 2007 till 2011. Both patients with suprapubic and transurethral catheters were eligible for inclusion. Notably, patients with sepsis were excluded. Sixty-one patients were randomized to receive either the 5-day (33 patients) or the 10-day (28 patients) regimen. The non-inferiority criteria for clinical cure were met in the per-protocol analysis, clinical cure at end of therapy was 100% in both groups. Microbiological response was lower in the replacement group (82.1% vs. 88.9%) at end of therapy. The 5-day regimen with catheter replacement was also associated with a higher incidence of recurrence compared to the 10-day regimen with catheter retainment (32.1% vs. 11.1%).

More recently, in 2018 Babich and coworkers published the results of their prospective, observational, cohort study conducted in six internal medicine departments and a department of geriatrics of a medical center [10]. Of the 315 CAUTI patients enrolled between 2010 and 2015, 98 had their transurethral catheter replaced, and 217 did not. In 16/98 patients in whom catheter was replaced, there was suspicion of obstruction or malposition. When data were analyzed without any adjustment for confounding, catheter replacement was associated with lower risk of clinical failure. However, after propensity score matching to adjust for confounding, there was no statistically significant association between catheter replacement and clinical failure (OR 0.90, 95%CI 0.50–1.63) or 30-day mortality (OR 0.76, 95%CI 0.40–1.44). Rehospitalization rates, including those for sepsis, were also similar in the replacement and retainment group. Antimicrobial treatment (adequacy/duration) was not included as a confounder in the analyses.

### Risk of bias assessments

A summary of the risk of bias assessments for the includes studies is presented in supplement S3. According to the Newcastle-Ottawa Scale, the study by Kumazawa et al. has a high risk of bias. Thesecond observational study by Babich scores a low risk of bias on most categories, however important confounders, such as antimicrobial treatment (adequacy and duration) were not included in the matching procedure The two RCT’s have a high risk of bias as a result of the open label design. In the study by Darouiche et al., an important risk of bias is introduced as a result of a 5-day difference in duration of antibiotic treatment between the catheter replacement group and catheter retainment group.

## Discussion

This systematic literature review identified four articles that investigated the effect of catheter replacement in patients with CAUTI. The two observational studies showed a neutral effect of catheter replacement [9, 10]. The two RCT’s had contradicting results [7, 8]. One RCT favoured catheter replacement, demonstrating higher rates of clinical cure, and lower recurrence rates in the replacement group [7]. The other RCT favoured retainment, with a lower recurrence rate in the retainment group [8]. 

Notably, out of the four identified studies only one study endorses catheter replacement as a component of CAUTI therapy [7]. This study provided the basis for the current guideline recommendations for catheter replacement in patients with CAUTI. The study suggests a substantial benefit of catheter replacement on both time to symptom resolution and recurrence rate. The difference with the other three studies may be explained by the generally low sample sizes and the heterogeneity in patient populations, definitions of CAUTI, antimicrobial treatment strategies and outcome assessments.

The overarching limitation lies in the modest scale of these studies. Both RCT’s have small sample sizes and the observational studies exhibit various methodological challenges and potential biases, of which (non-measured) confounding is the most important one. For example, doctors may be more inclined to change the catheter if the patient is severely ill or has had recurrent infections in the past. In all studies, misclassification of CAUTI is a potential risk both in the inclusion criteria and in defining recurrences. Diagnosing CAUTI is notoriously difficult, which is reflected by the different definitions used in the four studies. Asymptomatic bacteriuria may be erroneously diagnosed as CAUTI. For example, in the study by Kumazawa, the diagnosis was based on bacteriuria and pyuria, and symptoms were not specified. In the study of Raz et al., systemic symptoms were required for diagnosis, but no other investigations were performed to exclude alternative causes. In five patients of this study, urine cultures were already negative before start of treatment, which would not be expected in CAUTI. In two studies, either dosing or length of antibiotic therapy is a confounding factor. However, in the study by Kumazawa, levofloxacin dosage was lower in the retainment group, and therefore is not expected to impact the finding that catheter replacement did not improve clinical outcome. This is different for the RCT by Darouiche, where patients that were randomized to retainment of the catheter, received a longer duration of antibiotics, which may mask an effect of catheter replacement.

There are limitations to the current review. Firstly, there is the inherent risk of not capturing all relevant literature, despite efforts to conduct a comprehensive search. Additionally, reliance on the information provided in the selected studies introduces the potential for information bias. While the review benefitted from the expertise of a librarian and involved a duplicate screening process to enhance reliability, the human element in interpretation remains.

On theoretical grounds, catheter replacement is rational. It aligns with the general principle of source control in infectious disease management, aiming to eliminate or reduce the source of infection to optimize patient outcomes. Removing the source, i.e., the catheter, could theoretically accelerate symptom resolution. Furthermore, biofilms form on catheters, and pathogens can persists in these biofilms despite treatment [12]. Therefore, removing the catheter – and with it the biofilm – may prevent recurrences. Furthermore, biofilms can act as a reservoir for antibiotic resistance genes, and retaining the catheter combined with the selection pressure of antimicrobial therapy, may lead to the development of multidrug-resistant pathogens. Despite the theoretical basis for catheter replacement in CAUTI, empirical evidence from studies has not yet satisfactorily substantiated its efficacy. Of note, in case of catheter obstruction or malposition a replacement is always indicated, but in case of a adequate drainage, it remains unclear whether or not the catheter should be replaced.

Catheter replacement is not without burden [13]. It causes discomfort in patients, has a risk of complications and an impact on healthcare resources [14]. This is the case for transurethral catheters and suprapubic catheters, and the burden is even higher in JJ-catheters and nephrostomy catheters. For the latter category of patients there is not one study to assess the effect of a catheter change on symptom resolution or recurrence. Beyond the immediate health implications, catheter replacement contributes to the consumption of medical materials and has an environmental impact within the context of sustainable development goals (SDGs).

### Future trials

Based on this review, it is clear that a new trial is necessary, incorporating the lessons learned from the included studies. A randomized design is required because even with propensity score matching, the risk of bias due to unmeasured confounding persists. Secondly, the patient population should be well-defined. No definition is perfect, but using the IDSA definition of CAUTI will limit misclassification and enhance comparability across studies in the CAUTI field [1]. Patients for whom catheter replacement is non-debatable, such as those with catheter malfunction, should be excluded. The intervention should focus solely on the retention or replacement of the catheter, with antimicrobial therapy being consistent between both groups.

Outcome parameters should include both the clinical course of the initial CAUTI (for example time to resolution of symptoms) and relapse of CAUTI, as these are relevant on theoretical grounds and have shown effects in the study by Raz. Additionally, patient-related outcome measures, such as quality of life metrics, should be included. Outcome parameters should be established in collaboration with both patients and healthcare professionals. Sample size should be based on the ability to detect relevant clinical differences, as established by involving different stakeholders, including patients. Given that opinions on catheter replacement differ among specialties (e.g., urology versus microbiology), multidisciplinary collaboration in both the design and execution of the study is essential. This approach will ensure that the study results are incorporated into the guidelines of various professional associations and can find their way to clinical practice.

## Conclusion

The existing evidence regarding the necessity of catheter replacement in CAUTI is limited, despite its common occurrence. [1, 15] The current guideline recommendation for indwelling catheter replacement in patients with CAUTI is based on the findings of a solitary small-scale RCT, lacking confirmation in other studies. The burden of catheter replacement, including patient discomfort and resource impact, warrants careful consideration. A new randomized trial is essential to provide more evidence base on the effect of catheter replacement on clinical cure and recurrence rates in CAUTI patients.

### Electronic supplementary material

Below is the link to the electronic supplementary material.

Supplementary Material 1

Supplementary Material 2

Supplementary Material 3

Supplementary Material 4

## Data Availability

Data generated during this study will be shared on request.
