# Supplementary material for: “Catheter replacement in catheter-associated urinary tract infection: current state of evidence “
Source: Eur J Clin Microbiol Infect Dis. 2024 Jun 25;43(8):1631–7. doi: 10.1007/s10096-024-04878-9 (PMC11271365; doi:10.1007/s10096-024-04878-9)
Supplement: Supplementary file 1 — Supplementary Material 1 [file 10096_2024_4878_MOESM1_ESM.pdf]

Supplementary material 1. Search Strategy

Databases:

PubMed

(("Catheters, Indwelling"[Mesh] OR "indwelling catheter"[tw] OR "indwelling catheters"[tw] OR "in-dwelling catheter"[tw] OR "in-dwelling catheters"[tw] OR "Implantable Catheters"[tw] OR "Implantable Catheter"[tw] OR "Urinary Catheters"[mesh] OR "Urinary Catheters"[tw] OR "Urinary Catheter"[tw] OR "urethral catheter"[tw] OR "urethral catheters"[tw] OR "double j catheter"[tw] OR "jj catheter"[tw] OR "suprapubic catheter"[tw] OR "nephrostomy catheter"[tw] OR "bladder catheter"[tw] OR "double j catheters"[tw] OR "j catheters"[tw] OR "suprapubic catheters"[tw] OR "nephrostomy catheters"[tw] OR "bladder catheters"[tw] OR "indwelling catheter"[title/abstract:~4] OR "indwelling catheters"[title/abstract:~4] OR "in-dwelling catheter"[title/abstract:~4] OR "in-dwelling catheters"[title/abstract:~4] OR "Implantable Catheters"[title/abstract:~4] OR "Implantable Catheter"[title/abstract:~4] OR "Urinary Catheters"[title/abstract:~4] OR "Urinary Catheter"[title/abstract:~4] OR "urethral catheter"[title/abstract:~4] OR "urethral catheters"[title/abstract:~4] OR "double j catheter"[title/abstract:~4] OR "jj catheter"[title/abstract:~4] OR "suprapubic catheter"[title/abstract:~4] OR "nephrostomy catheter"[title/abstract:~4] OR "bladder catheter"[title/abstract:~4] OR "double j catheters"[title/abstract:~4] OR "j catheters"[title/abstract:~4] OR "suprapubic catheters"[title/abstract:~4] OR "nephrostomy catheters"[title/abstract:~4] OR "bladder catheters"[title/abstract:~4] OR "Catheters"[majr] OR "catheter"[ti] OR "catheters"[ti]) AND ("Urinary Tract Infections"[mesh] OR "Urinary Tract Infections"[tw] OR "Urinary Tract Infection"[tw] OR "Urinary Infections"[tw] OR "Urinary Infection"[tw] OR "Bacteriuria"[tw] OR "Bacteriuria*"[tw] OR "Pyuria"[tw] OR "Pyuria*"[tw] OR "Cystitis"[Mesh] OR "Cystitis"[tw] OR "bladder infection"[tw] OR "Pyelocystitis"[tw] OR "Pyelonephritis"[Mesh] OR "Pyelonephritis"[tw] OR "CAUTI"[tw] OR "CAUTIs"[tw] OR ("Catheter-Related Infections"[mesh] AND ("Urinary Tract"[mesh] OR "urinary"[tw])) OR "Urinary Tract Infections"[title/abstract:~4] OR "Urinary Tract Infection"[title/abstract:~4] OR "Urinary Infections"[title/abstract:~4] OR "Urinary Infection"[title/abstract:~4] OR "bladder infection"[title/abstract:~4]) AND ("catheter replacement"[title/abstract:~3] OR "catheters replacements"[title/abstract:~3] OR "catheter replacement"[title/abstract:~3] OR "catheters replacements"[title/abstract:~3] OR "catheter removal "[title/abstract:~3] OR "catheters removal "[title/abstract:~3] OR "Device Removal"[Mesh] OR "switch catheter"[title/abstract:~3] OR "switched catheter"[title/abstract:~3] OR "switches catheter"[title/abstract:~3] OR "switching catheter"[title/abstract:~3] OR "switch catheters"[title/abstract:~3] OR "switched catheters"[title/abstract:~3] OR "switches catheters"[title/abstract:~3] OR "switching catheters"[title/abstract:~3] OR "change catheter"[title/abstract:~3] OR "changed catheter"[title/abstract:~3] OR "changes catheter"[title/abstract:~3] OR "changing

catheter"[title/abstract:~3] OR "change catheters"[title/abstract:~3] OR "changed catheters"[title/abstract:~3] OR "changes catheters"[title/abstract:~3] OR "changing catheters"[title/abstract:~3] **OR "catheter exchange"[title/abstract:~3] OR "catheter exchanges"[title/abstract:~3] OR "catheter exchanged"[title/abstract:~3]**) AND ("Recurrence"[Mesh] OR "Recurrence"[tw] OR "Recurr*"[tw] OR "reinfection"[tw] OR "reinfect*"[tw] OR "re-infection"[tw] OR "re-infect"[tw] OR "relapse"[tw] OR "relaps*"[tw] OR "duration complaint"[title/abstract:~3] OR "duration complaints"[title/abstract:~3] OR "duration symptom"[title/abstract:~3] OR "duration symptoms"[title/abstract:~3] OR "duration sign"[title/abstract:~3] OR "duration signs"[title/abstract:~3] OR "duration infection"[title/abstract:~3] OR "duration infections"[title/abstract:~3] OR (("complaint"[tw] OR "complaints"[tw] OR "symptom"[tw] OR "symptoms"[tw] OR "sign"[tw] OR "signs"[tw] OR "Signs and Symptoms"[Mesh:NoExp] OR "Urological Manifestations"[Mesh] **OR "outcome"[tw] OR "outcomes"[tw]**) AND ("duration"[tw] OR "Time Factors"[mesh])) **OR "Urinary Tract Infections/complications"[Mesh]**) NOT ("Animals"[mesh] NOT "Humans"[mesh]))

Indwelling/Urinary Catheters & Urinary Tract Infections & Catheter replacement & Recurrence/Reinfection/Duration of complaints

(("Catheters, Indwelling"[Mesh] OR "indwelling catheter"[tw] OR "indwelling catheters"[tw] OR "in-dwelling catheter"[tw] OR "in-dwelling catheters"[tw] OR "Implantable Catheters"[tw] OR "Implantable Catheter"[tw] OR "Urinary Catheters"[mesh] OR "Urinary Catheters"[tw] OR "Urinary Catheter"[tw] OR "urethral catheter"[tw] OR "urethral catheters"[tw] OR "double j catheter"[tw] OR "jj catheter"[tw] OR "suprapubic catheter"[tw] OR "nephrostomy catheter"[tw] OR "bladder catheter"[tw] OR "double j catheters"[tw] OR "j catheters"[tw] OR "suprapubic catheters"[tw] OR "nephrostomy catheters"[tw] OR "bladder catheters"[tw] OR "indwelling catheter"[title/abstract:~4] OR "indwelling catheters"[title/abstract:~4] OR "in-dwelling catheter"[title/abstract:~4] OR "in-dwelling catheters"[title/abstract:~4] OR "Implantable Catheters"[title/abstract:~4] OR "Implantable Catheter"[title/abstract:~4] OR "Urinary Catheters"[title/abstract:~4] OR "Urinary Catheter"[title/abstract:~4] OR "urethral catheter"[title/abstract:~4] OR "urethral catheters"[title/abstract:~4] OR "double j catheter"[title/abstract:~4] OR "jj catheter"[title/abstract:~4] OR "suprapubic catheter"[title/abstract:~4] OR "nephrostomy catheter"[title/abstract:~4] OR "bladder catheter"[title/abstract:~4] OR "double j catheters"[title/abstract:~4] OR "j catheters"[title/abstract:~4] OR "suprapubic catheters"[title/abstract:~4] OR "nephrostomy catheters"[title/abstract:~4] OR "bladder catheters"[title/abstract:~4] OR "Catheters"[majr] OR "catheter"[ti] OR "catheters"[ti]) AND ("Urinary Tract Infections"[mesh] OR "Urinary Tract Infections"[tw] OR "Urinary Tract Infection"[tw] OR "Urinary Infections"[tw] OR "Urinary Infection"[tw] OR "Bacteriuria"[tw] OR "Bacteriuria*"[tw] OR "Pyuria"[tw] OR "Pyuria*"[tw] OR "Cystitis"[Mesh] OR "Cystitis"[tw] OR "bladder infection"[tw] OR "Pyelocystitis"[tw] OR "Pyelonephritis"[Mesh] OR "Pyelonephritis"[tw] OR "CAUTI"[tw] OR "CAUTIs"[tw] OR ("Catheter-Related Infections"[mesh] AND ("Urinary Tract"[mesh] OR "urinary"[tw])) OR "Urinary Tract Infections"[title/abstract:~4] OR "Urinary Tract Infection"[title/abstract:~4] OR "Urinary Infections"[title/abstract:~4] OR "Urinary Infection"[title/abstract:~4] OR "bladder infection"[title/abstract:~4]) AND ("catheter replacement"[title/abstract:~3] OR "catheters replacements"[title/abstract:~3] OR "catheter replacement"[title/abstract:~3] OR "catheters replacements"[title/abstract:~3] OR "catheter removal "[title/abstract:~3] OR "catheters removal "[title/abstract:~3] OR "Device Removal"[Mesh] OR "switch catheter"[title/abstract:~3] OR "switched catheter"[title/abstract:~3] OR "switches catheter"[title/abstract:~3] OR "switching catheter"[title/abstract:~3] OR "switch catheters"[title/abstract:~3] OR "switched

catheters"[title/abstract:~3] OR "switches catheters"[title/abstract:~3] OR "switching catheters"[title/abstract:~3] OR "change catheter"[title/abstract:~3] OR "changed catheter"[title/abstract:~3] OR "changes catheter"[title/abstract:~3] OR "changing catheter"[title/abstract:~3] OR "change catheters"[title/abstract:~3] OR "changed catheters"[title/abstract:~3] OR "changes catheters"[title/abstract:~3] OR "changing catheters"[title/abstract:~3]) AND ("Recurrence"[Mesh] OR "Recurrence"[tw] OR "Recurr*"[tw] OR "reinfection"[tw] OR "reinfect*"[tw] OR "re-infection"[tw] OR "re-infect"[tw] OR "relapse"[tw] OR "relaps*"[tw] OR "duration complaint"[title/abstract:~3] OR "duration complaints"[title/abstract:~3] OR "duration symptom"[title/abstract:~3] OR "duration symptoms"[title/abstract:~3] OR "duration sign"[title/abstract:~3] OR "duration signs"[title/abstract:~3] OR "duration infection"[title/abstract:~3] OR "duration infections"[title/abstract:~3] OR (("complaint"[tw] OR "complaints"[tw] OR "symptom"[tw] OR "symptoms"[tw] OR "sign"[tw] OR "signs"[tw] OR "Signs and Symptoms"[Mesh:NoExp] OR "Urological Manifestations"[Mesh]) AND ("duration"[tw] OR "Time Factors"[mesh]))) NOT ("Animals"[mesh] NOT "Humans"[mesh]))

Embase
http://ovidsp.ovid.com/ovidweb.cgi?T=JS&PAGE=main&MODE=ovid&D=oemezd

((exp *"Indwelling Catheter"/ OR "indwelling catheter".ti,ab OR "indwelling catheters".ti,ab OR "in-dwelling catheter".ti,ab OR "in-dwelling catheters".ti,ab OR "Implantable Catheters".ti,ab OR "Implantable Catheter".ti,ab OR exp *"Urinary Catheter"/ OR "Urinary Catheters".ti,ab OR "Urinary Catheter".ti,ab OR "urethral catheter".ti,ab OR "urethral catheters".ti,ab OR "double j catheter".ti,ab OR "jj catheter".ti,ab OR exp *"suprapubic catheter"/ OR "suprapubic catheter".ti,ab OR "nephrostomy catheter".ti,ab OR "bladder catheter".ti,ab OR "double j catheters".ti,ab OR "j catheters".ti,ab OR "suprapubic catheters".ti,ab OR "nephrostomy catheters".ti,ab OR "bladder catheters".ti,ab OR (("indwelling" ADJ4 "catheter") OR ("indwelling" ADJ4 "catheters") OR ("in-dwelling" ADJ4 "catheter") OR ("in-dwelling" ADJ4 "catheters") OR ("Implantable" ADJ4 "Catheters") OR ("Implantable" ADJ4 "Catheter") OR ("Urinary" ADJ4 "Catheters") OR ("Urinary" ADJ4 "Catheter") OR ("urethral" ADJ4 "catheter") OR ("urethral" ADJ4 "catheters") OR ("double" ADJ4 "j" ADJ4 "catheter") OR ("jj" ADJ4 "catheter") OR ("suprapubic" ADJ4 "catheter") OR ("nephrostomy" ADJ4 "catheter") OR ("bladder" ADJ4 "catheter") OR ("double" ADJ4 "j" ADJ4 "catheters") OR ("j" ADJ4 "catheters") OR ("suprapubic" ADJ4 "catheters") OR ("nephrostomy" ADJ4 "catheters") OR ("bladder" ADJ4 "catheters")).ti,ab OR exp *"Catheter"/ OR "catheter".ti OR "catheters".ti) AND (exp *"Urinary Tract Infection"/ OR "Urinary Tract Infections".ti,ab OR "Urinary Tract Infection".ti,ab OR "Urinary Infections".ti,ab OR "Urinary Infection".ti,ab OR "Bacteriuria".ti,ab OR "Bacteriuria*".ti,ab OR "Pyuria".ti,ab OR "Pyuria*".ti,ab OR exp *"Cystitis"/ OR "Cystitis".ti,ab OR "bladder infection".ti,ab OR "Pyelocystitis".ti,ab OR exp *"Pyelonephritis"/ OR "Pyelonephritis".ti,ab OR "CAUTI".ti,ab OR "CAUTIs".ti,ab OR (exp *"Catheter Infection"/ AND ("exp *Urinary Tract"/ OR "urinary".ti,ab)) OR (("Urinary" ADJ4 "Tract" ADJ4 "Infections") OR ("Urinary" ADJ4 "Tract" ADJ4 "Infection") OR ("Urinary" ADJ4 "Infections") OR ("Urinary" ADJ4 "Infection") OR ("bladder" ADJ4 "infection")).ti,ab) AND (exp *"catheter removal"/ OR exp *"Device Removal"/ OR (("catheter" ADJ3 "replacement") OR ("catheters" ADJ3 "replacements") OR ("catheter" ADJ3 "replacement") OR ("catheters" ADJ3 "replacements") OR ("catheter" ADJ3 "removal") OR ("catheters" ADJ3 "removal") OR ("switch" ADJ3 "catheter") OR ("switched" ADJ3 "catheter") OR ("switches" ADJ3 "catheter") OR ("switching" ADJ3 "catheter") OR ("switch" ADJ3

"catheters") OR ("switched" ADJ3 "catheters") OR ("switches" ADJ3 "catheters") OR ("switching" ADJ3 "catheters") OR ("change" ADJ3 "catheter") OR ("changed" ADJ3 "catheter") OR ("changes" ADJ3 "catheter") OR ("changing" ADJ3 "catheter") OR ("change" ADJ3 "catheters") OR ("changed" ADJ3 "catheters") OR ("changes" ADJ3 "catheters") OR ("changing" ADJ3 "catheters") **OR ("catheter" ADJ3 "exchange") OR ("catheter" ADJ3 "exchanges") OR ("catheter" ADJ3 "exchanged")**).ti,ab) AND (exp "Recurrent Disease"/ OR "reinfection"/ OR "Recurrence".ti,ab OR "Recurr*".ti,ab OR "reinfection".ti,ab OR "reinfect*".ti,ab OR "re-infection".ti,ab OR "re-infect".ti,ab OR "relapse".ti,ab OR "relaps*".ti,ab OR (("duration" ADJ3 "complaint") OR ("duration" ADJ3 "complaints") OR ("duration" ADJ3 "symptom") OR ("duration" ADJ3 "symptoms") OR ("duration" ADJ3 "sign") OR ("duration" ADJ3 "signs") OR ("duration" ADJ3 "infection") OR ("duration" ADJ3 "infections")).ti,ab OR (("complaint".ti,ab OR "complaints".ti,ab OR "symptom".ti,ab OR "symptoms".ti,ab OR "sign".ti,ab OR "signs".ti,ab OR exp "lower urinary tract symptom"/ **OR "outcome".mp OR "outcomes".mp**) AND ("duration".ti,ab OR exp "Time Factor"/)) **OR exp *"Urinary Tract Infection"/co**) NOT (exp "Animals"/ NOT exp "Humans"/))

- NOT conference review.pt
- NOT (conference abstract).pt

Web of Science
http://isiknowledge.com/wos

((TS=("Indwelling Catheter" OR "indwelling catheter" OR "indwelling catheters" OR "in-dwelling catheter" OR "in-dwelling catheters" OR "Implantable Catheters" OR "Implantable Catheter" OR "Urinary Catheter" OR "Urinary Catheters" OR "Urinary Catheter" OR "urethral catheter" OR "urethral catheters" OR "double j catheter" OR "jj catheter" OR "suprapubic catheter" OR "suprapubic catheter" OR "nephrostomy catheter" OR "bladder catheter" OR "double j catheters" OR "j catheters" OR "suprapubic catheters" OR "nephrostomy catheters" OR "bladder catheters" OR (("indwelling" NEAR/4 "catheter") OR ("indwelling" NEAR/4 "catheters") OR ("in-dwelling" NEAR/4 "catheter") OR ("in-dwelling" NEAR/4 "catheters") OR ("Implantable" NEAR/4 "Catheters") OR ("Implantable" NEAR/4 "Catheter") OR ("Urinary" NEAR/4 "Catheters") OR ("Urinary" NEAR/4 "Catheter") OR ("urethral" NEAR/4 "catheter") OR ("urethral" NEAR/4 "catheters") OR ("double" NEAR/4 "j" NEAR/4 "catheter") OR ("jj" NEAR/4 "catheter") OR ("suprapubic" NEAR/4 "catheter") OR ("nephrostomy" NEAR/4 "catheter") OR ("bladder" NEAR/4 "catheter") OR ("double" NEAR/4 "j" NEAR/4 "catheters") OR ("j" NEAR/4 "catheters") OR ("suprapubic" NEAR/4 "catheters") OR ("nephrostomy" NEAR/4 "catheters") OR ("bladder" NEAR/4 "catheters"))) OR TI=("catheter" OR "catheters")) AND TS=("Urinary Tract Infection" OR "Urinary Tract Infections" OR "Urinary Tract Infection" OR "Urinary Infections" OR "Urinary Infection" OR "Bacteriuria" OR "Bacteriuria*" OR "Pyuria" OR "Pyuria*" OR "Cystitis" OR "Cystitis" OR "bladder infection" OR "Pyelocystitis" OR "Pyelonephritis" OR "Pyelonephritis" OR "CAUTI" OR "CAUTIs" OR ("Catheter Infection" AND ("Urinary Tract" OR "urinary")) OR (("Urinary" NEAR/4 "Tract" NEAR/4 "Infections") OR ("Urinary" NEAR/4 "Tract" NEAR/4 "Infection") OR ("Urinary" NEAR/4 "Infections")

OR ("Urinary" NEAR/4 "Infection") OR ("bladder" NEAR/4 "infection"))) AND TS=("catheter removal" OR "Device Removal" OR (("catheter" NEAR/3 "replacement") OR ("catheters" NEAR/3 "replacements") OR ("catheter" NEAR/3 "replacement") OR ("catheters" NEAR/3 "replacements") OR ("catheter" NEAR/3 "removal") OR ("catheters" NEAR/3 "removal") OR ("switch" NEAR/3 "catheter") OR ("switched" NEAR/3 "catheter") OR ("switches" NEAR/3 "catheter") OR ("switching" NEAR/3 "catheter") OR ("switch" NEAR/3 "catheters") OR ("switched" NEAR/3 "catheters") OR ("switches" NEAR/3 "catheters") OR ("switching" NEAR/3 "catheters") OR ("change" NEAR/3 "catheter") OR ("changed" NEAR/3 "catheter") OR ("changes" NEAR/3 "catheter") OR ("changing" NEAR/3 "catheter") OR ("change" NEAR/3 "catheters") OR ("changed" NEAR/3 "catheters") OR ("changes" NEAR/3 "catheters") OR ("changing" NEAR/3 "catheters") **OR ("catheter" NEAR/3 "exchange") OR ("catheter" NEAR/3 "exchanges") OR ("catheter" NEAR/3 "exchanged")**)) AND TS=("Recurrent Disease" OR "reinfection" OR "Recurrence" OR "Recurr*" OR "reinfection" OR "reinfect*" OR "re-infection" OR "re-infect" OR "relapse" OR "relaps*" OR (("duration" NEAR/3 "complaint") OR ("duration" NEAR/3 "complaints") OR ("duration" NEAR/3 "symptom") OR ("duration" NEAR/3 "symptoms") OR ("duration" NEAR/3 "sign") OR ("duration" NEAR/3 "signs") OR ("duration" NEAR/3 "infection") OR ("duration" NEAR/3 "infections")) OR (("complaint" OR "complaints" OR "symptom" OR "symptoms" OR "sign" OR "signs" OR "lower urinary tract symptom" **OR "outcome" OR "outcomes"**) AND ("duration" OR "Time Factor"))) NOT TI=("veterinary" OR "rabbit" OR "rabbits" OR "animal" OR "animals" OR "mouse" OR "mice" OR "rodent" OR "rodents" OR "rat" OR "rats" OR "pig" OR "pigs" OR "porcine" OR "horse" OR "horses" OR "equine" OR "cow" OR "cows" OR "bovine" OR "goat" OR "goats" OR "sheep" OR "ovine" OR "canine" OR "dog" OR "dogs" OR "feline" OR "cat" OR "cats"))

Cochrane
https://www.cochranelibrary.com/advanced-search/search-manager

((("Indwelling Catheter" OR "indwelling catheter" OR "indwelling catheters" OR "in-dwelling catheter" OR "in-dwelling catheters" OR "Implantable Catheters" OR "Implantable Catheter" OR "Urinary Catheter" OR "Urinary Catheters" OR "Urinary Catheter" OR "urethral catheter" OR "urethral catheters" OR "double j catheter" OR "jj catheter" OR "suprapubic catheter" OR "suprapubic catheter" OR "nephrostomy catheter" OR "bladder catheter" OR "double j catheters" OR "j catheters" OR "suprapubic catheters" OR "nephrostomy catheters" OR "bladder catheters" OR (("indwelling" NEAR/4 "catheter") OR ("indwelling" NEAR/4 "catheters") OR ("in-dwelling" NEAR/4 "catheter") OR ("in-dwelling" NEAR/4 "catheters") OR ("Implantable" NEAR/4 "Catheters") OR ("Implantable" NEAR/4 "Catheter") OR ("Urinary" NEAR/4 "Catheters") OR ("Urinary" NEAR/4 "Catheter") OR ("urethral" NEAR/4 "catheter") OR ("urethral" NEAR/4 "catheters") OR ("double" NEAR/4 "j" NEAR/4 "catheter") OR ("jj" NEAR/4 "catheter") OR ("suprapubic" NEAR/4 "catheter") OR ("nephrostomy" NEAR/4 "catheter") OR ("bladder" NEAR/4 "catheter") OR ("double" NEAR/4 "j" NEAR/4 "catheters") OR ("j" NEAR/4 "catheters") OR ("suprapubic" NEAR/4 "catheters") OR ("nephrostomy" NEAR/4 "catheters") OR ("bladder" NEAR/4 "catheters"))):ti,ab,kw OR

("catheter" OR "catheters"):ti) AND ("Urinary Tract Infection" OR "Urinary Tract Infections" OR "Urinary Tract Infection" OR "Urinary Infections" OR "Urinary Infection" OR "Bacteriuria" OR "Bacteriuria*" OR "Pyuria" OR "Pyuria*" OR "Cystitis" OR "Cystitis" OR "bladder infection" OR "Pyelocystitis" OR "Pyelonephritis" OR "Pyelonephritis" OR "CAUTI" OR "CAUTIs" OR ("Catheter Infection" AND ("Urinary Tract" OR "urinary")) OR (("Urinary" NEAR/4 "Tract" NEAR/4 "Infections") OR ("Urinary" NEAR/4 "Tract" NEAR/4 "Infection") OR ("Urinary" NEAR/4 "Infections") OR ("Urinary" NEAR/4 "Infection") OR ("bladder" NEAR/4 "infection"))):ti,ab,kw AND ("catheter removal" OR "Device Removal" OR (("catheter" NEAR/3 "replacement") OR ("catheters" NEAR/3 "replacements") OR ("catheter" NEAR/3 "replacement") OR ("catheters" NEAR/3 "replacements") OR ("catheter" NEAR/3 "removal") OR ("catheters" NEAR/3 "removal") OR ("switch" NEAR/3 "catheter") OR ("switched" NEAR/3 "catheter") OR ("switches" NEAR/3 "catheter") OR ("switching" NEAR/3 "catheter") OR ("switch" NEAR/3 "catheters") OR ("switched" NEAR/3 "catheters") OR ("switches" NEAR/3 "catheters") OR ("switching" NEAR/3 "catheters") OR ("change" NEAR/3 "catheter") OR ("changed" NEAR/3 "catheter") OR ("changes" NEAR/3 "catheter") OR ("changing" NEAR/3 "catheter") OR ("change" NEAR/3 "catheters") OR ("changed" NEAR/3 "catheters") OR ("changes" NEAR/3 "catheters") OR ("changing" NEAR/3 "catheters") **OR ("catheter" NEAR/3 "exchange") OR ("catheter" NEAR/3 "exchanges") OR ("catheter" NEAR/3 "exchanged")**))):ti,ab,kw AND ("Recurrent Disease" OR "reinfection" OR "Recurrence" OR "Recurr*" OR "reinfection" OR "reinfect*" OR "re-infection" OR "re-infect" OR "relapse" OR "relaps*" OR (("duration" NEAR/3 "complaint") OR ("duration" NEAR/3 "complaints") OR ("duration" NEAR/3 "symptom") OR ("duration" NEAR/3 "symptoms") OR ("duration" NEAR/3 "sign") OR ("duration" NEAR/3 "signs") OR ("duration" NEAR/3 "infection") OR ("duration" NEAR/3 "infections")) OR (("complaint" OR "complaints" OR "symptom" OR "symptoms" OR "sign" OR "signs" OR "lower urinary tract symptom" **OR "outcome" OR "outcomes"**) AND ("duration" OR "Time Factor"))):ti,ab,kw)

(conference abstract OR meeting abstract OR conference proceeding OR conference proceedings):pt

Emcare
http://ovidsp.ovid.com/ovidweb.cgi?T=JS&NEWS=n&CSC=Y&PAGE=main&D=emcr

((exp *"Indwelling Catheter"/ OR "indwelling catheter".ti,ab OR "indwelling catheters".ti,ab OR "in-dwelling catheter".ti,ab OR "in-dwelling catheters".ti,ab OR "Implantable Catheters".ti,ab OR "Implantable Catheter".ti,ab OR exp *"Urinary Catheter"/ OR "Urinary Catheters".ti,ab OR "Urinary Catheter".ti,ab OR "urethral catheter".ti,ab OR "urethral catheters".ti,ab OR "double j catheter".ti,ab OR "jj catheter".ti,ab OR exp *"suprapubic catheter"/ OR "suprapubic catheter".ti,ab OR "nephrostomy catheter".ti,ab OR "bladder catheter".ti,ab OR "double j catheters".ti,ab OR "j catheters".ti,ab OR "suprapubic catheters".ti,ab OR "nephrostomy catheters".ti,ab OR "bladder catheters".ti,ab OR (("indwelling" ADJ4 "catheter") OR ("indwelling" ADJ4 "catheters") OR ("in-dwelling" ADJ4 "catheter") OR ("in-dwelling" ADJ4 "catheters") OR ("Implantable" ADJ4 "Catheters") OR ("Implantable" ADJ4 "Catheter") OR

("Urinary" ADJ4 "Catheters") OR ("Urinary" ADJ4 "Catheter") OR ("urethral" ADJ4 "catheter") OR ("urethral" ADJ4 "catheters") OR ("double" ADJ4 "j" ADJ4 "catheter") OR ("jj" ADJ4 "catheter") OR ("suprapubic" ADJ4 "catheter") OR ("nephrostomy" ADJ4 "catheter") OR ("bladder" ADJ4 "catheter") OR ("double" ADJ4 "j" ADJ4 "catheters") OR ("j" ADJ4 "catheters") OR ("suprapubic" ADJ4 "catheters") OR ("nephrostomy" ADJ4 "catheters") OR ("bladder" ADJ4 "catheters")).ti,ab OR exp *"Catheter"/ OR "catheter".ti OR "catheters".ti) AND (exp *"Urinary Tract Infection"/ OR "Urinary Tract Infections".ti,ab OR "Urinary Tract Infection".ti,ab OR "Urinary Infections".ti,ab OR "Urinary Infection".ti,ab OR "Bacteriuria".ti,ab OR "Bacteriuria*".ti,ab OR "Pyuria".ti,ab OR "Pyuria*".ti,ab OR exp *"Cystitis"/ OR "Cystitis".ti,ab OR "bladder infection".ti,ab OR "Pyelocystitis".ti,ab OR exp *"Pyelonephritis"/ OR "Pyelonephritis".ti,ab OR "CAUTI".ti,ab OR "CAUTIs".ti,ab OR (exp *"Catheter Infection"/ AND ("exp *Urinary Tract"/ OR "urinary".ti,ab)) OR (("Urinary" ADJ4 "Tract" ADJ4 "Infections") OR ("Urinary" ADJ4 "Tract" ADJ4 "Infection") OR ("Urinary" ADJ4 "Infections") OR ("Urinary" ADJ4 "Infection") OR ("bladder" ADJ4 "infection")).ti,ab) AND (exp *"catheter removal"/ OR exp *"Device Removal"/ OR (("catheter" ADJ3 "replacement") OR ("catheters" ADJ3 "replacements") OR ("catheter" ADJ3 "replacement") OR ("catheters" ADJ3 "replacements") OR ("catheter" ADJ3 "removal") OR ("catheters" ADJ3 "removal") OR ("switch" ADJ3 "catheter") OR ("switched" ADJ3 "catheter") OR ("switches" ADJ3 "catheter") OR ("switching" ADJ3 "catheter") OR ("switch" ADJ3 "catheters") OR ("switched" ADJ3 "catheters") OR ("switches" ADJ3 "catheters") OR ("switching" ADJ3 "catheters") OR ("change" ADJ3 "catheter") OR ("changed" ADJ3 "catheter") OR ("changes" ADJ3 "catheter") OR ("changing" ADJ3 "catheter") OR ("change" ADJ3 "catheters") OR ("changed" ADJ3 "catheters") OR ("changes" ADJ3 "catheters") OR ("changing" ADJ3 "catheters") **OR ("catheter" ADJ3 "exchange") OR ("catheter" ADJ3 "exchanges") OR ("catheter" ADJ3 "exchanged")**).ti,ab) AND (exp "Recurrent Disease"/ OR "reinfection"/ OR "Recurrence".ti,ab OR "Recurr*".ti,ab OR "reinfection".ti,ab OR "reinfect*".ti,ab OR "re-infection".ti,ab OR "re-infect".ti,ab OR "relapse".ti,ab OR "relaps*".ti,ab OR (("duration" ADJ3 "complaint") OR ("duration" ADJ3 "complaints") OR ("duration" ADJ3 "symptom") OR ("duration" ADJ3 "symptoms") OR ("duration" ADJ3 "sign") OR ("duration" ADJ3 "signs") OR ("duration" ADJ3 "infection") OR ("duration" ADJ3 "infections")).ti,ab OR (("complaint".ti,ab OR "complaints".ti,ab OR "symptom".ti,ab OR "symptoms".ti,ab OR "sign".ti,ab OR "signs".ti,ab OR exp "lower urinary tract symptom"/ **OR "outcome".mp OR "outcomes".mp**) AND ("duration".ti,ab OR exp "Time Factor"/))) NOT (exp "Animals"/ NOT exp "Humans"/))
