# Supplementary material for: “Catheter replacement in catheter-associated urinary tract infection: current state of evidence “
Source: Eur J Clin Microbiol Infect Dis. 2024 Jun 25;43(8):1631–7. doi: 10.1007/s10096-024-04878-9 (PMC11271365; doi:10.1007/s10096-024-04878-9)
Supplement: Supplementary file 2 — Supplementary Material 2 [file 10096_2024_4878_MOESM2_ESM.docx]

**Supplementary material 2. PRISMA flowchart**

Studies from databases/registers **(n = 257)**

**Identification**

Studies included in review **(n = 4)**

Studies excluded **(n = 241)***

Studies assessed for eligibility **(n = 12)**

Studies screened **(n = 253)**

Studies excluded **(n = 8)**

Inappropriate intervention (n = 6)

Inapproriate patient population (n = 2)

e

References removed **(n = 4)**

Duplicates identified by Covidence (n = 4 )

**Screening**

**Included**

**Legend: PRISMA flowchart of study selection process***Large number of records involved catheter replacement for other indications
