# Supplementary material for: “Catheter replacement in catheter-associated urinary tract infection: current state of evidence “
Source: Eur J Clin Microbiol Infect Dis. 2024 Jun 25;43(8):1631–7. doi: 10.1007/s10096-024-04878-9 (PMC11271365; doi:10.1007/s10096-024-04878-9)
Supplement: Supplementary file 4 — Supplementary Material 4 [file 10096_2024_4878_MOESM4_ESM.docx]

**Supplementary material 3. Quality assessment**

| Study | Representativeness of the exposed cohort | Selection of the non-exposed cohort | Ascertainment of exposure | Demonstration that outcome of interest was not present at start of the study | Comparability of cohorts on the basis of the design or analyses | Assessment of outcome | Follow-up long enough for the outcome to occur | Adequacy of follow-up |
| --- | --- | --- | --- | --- | --- | --- | --- | --- |
| Kumazawa, 1992 | High risk of bias  *No description of the derivation of the cohort* | High risk of bias  *No description of the derivation of the cohort* | High risk of bias  *No description* | Not applicable | High risk of bias  *Different dose of and length of antimicrobial therapy, no corrections in the analyses.* | High risk of bias  *Assessment of outcome not reported* | High risk of bias  *Not reported* | High risk of bias  *No statement* |
| Babich  2018 | Low risk of bias  *Truly representative*  *Cohort of adults with transurethral catheters and CAUTI on internal medicine and geriatric wards* | Low risk of bias  *Drawn from the same community as the exposed cohort* | Low risk of bias  *Secure record* | Not applicable | Low risk of bias  *The study controls for age, sex, place of UTI acquisition, history of UTI, co-morbidities, antibiotic treatment, clinical symptoms* | Low risk of bias  *Record linkage* | Low risk of bias  *30 days* | Low risk of bias  *Complete* |

**Legend**: A summary of the risk of bias assessments for the two observational studies. Newcastle-Ottawa Scale. (1)

| Study | Bias arising from randomiation proces | Bias due to deviations from intended interventions | Bias in measurement of the outcome | Bias due to missing outcome data | Bias in selection of the reported results | Other Bias | Overall risk of bias |  |
| --- | --- | --- | --- | --- | --- | --- | --- | --- |
| Raz  2018 | Low risk of bias  *List developed by drawing lots*  *Allocation concealment not described* | Some concerns  *Open label, participants and professionals aware of treatment allocation. Deviations from allocation not described.* | High risk of bias  *Open label study* | Low risk of bias  *No lost to follow-up. Outcome data complete.* | Low risk of bias  *All prespecified outcomes were reported* | Not applicable | High risk of bias |  |
| Darouiche | Some concerns  *Randomization methods not described*  *Allocation concealment not described* | Some concerns  *Open label, participants and professionals aware of treatment allocation. Deviations from allocation not described.* | High risk of bias  *Open label study* | Low risk of bias  *Four patients lost to follow-up.* | Low risk of bias  *All prespecified outcomes were reported. Per protocol and intention modified intention to treat analyses* | *Differences in antibiotic treatment duration between experimental group and control group* | High risk of bias |  |

**Legend**: A summary of the risk of bias assessments for the two randomized trials. Cochrane Collaboration’s tool for assessing risk of bias in randomised trials. (2)

**References**

1. Wells GA, Shea B, O'Connell D, J. P, Welch V, Losos M, et al. The Newcastle-Ottawa Scale (NOS) for assessing the quality of nonrandomised studies in meta-analyses <http://www.ohri.ca/programs/clinical_epidemiology/oxford.asp2019> [

2. Sterne JAC, Savovic J, Page MJ, Elbers RG, Blencowe NS, Boutron I, et al. RoB 2: a revised tool for assessing risk of bias in randomised trials. BMJ. 2019;366:l4898.
